# Supplementary material for: Caffeic Acid Phenethyl Ester Protects Neurons Against Oxidative Stress and Neurodegeneration During Traumatic Brain Injury
Source: Biomolecules. 2025 Jan 8;15(1):80. doi: 10.3390/biom15010080 (PMC11762460; doi:10.3390/biom15010080)
Supplement: Supplementary file 1 [file biomolecules-15-00080-s001.zip › biomolecules-3385936-supplementary.pdf]

Supplementary figure

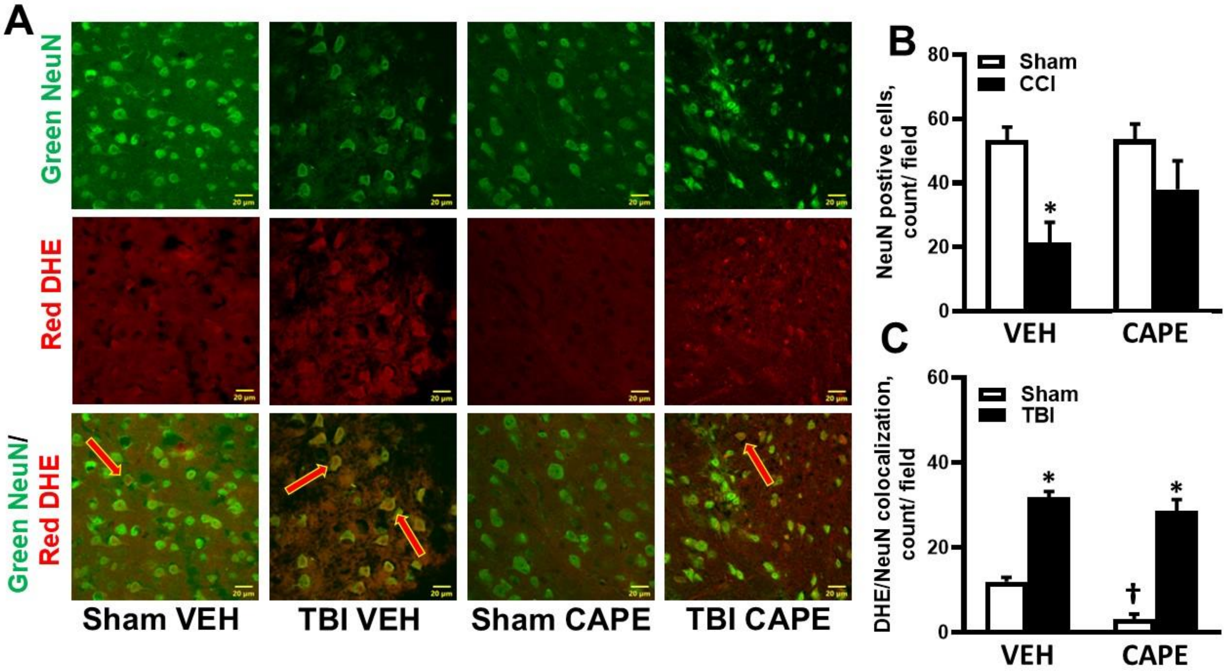

Figure S1. Neuronal degeneration 14 days after cortical contusion injury (CCI) in mice treated with caffeic acid phenethyl ester (CAPE) or vehicle (VEH). (**A**) Examples of the expression of neuronal marker, NeuN (green, upper row), generation of reactive oxygen species assessed by dihydroethidium (DHE) (red, middle row), and their co-localization seen as yellow dots (bottom row) and indicated by red arrows. Summaries of the count of (**B**) NeuN positive cells and their co-localization with DHE (**C**). $p < 0.05$; *-vs. Sham, †-vs. Sham VEH; n=6.
